# Airway Complications after Lung Transplantation—A Contemporary Series of 400 Bronchial Anastomoses from a Single Center

**DOI:** 10.3390/jcm12093061

**Published:** 2023-04-23

**Authors:** Anna Muñoz-Fos, Paula Moreno, Francisco Javier González, Eloisa Ruiz, Jose Manuel Vaquero, Carlos Baamonde, Francisco Cerezo, Javier Algar, Ricard Ramos-Izquierdo, Ángel Salvatierra, Antonio Alvarez

**Affiliations:** 1 Department of Thoracic Surgery, Bellvitge University Hospital, 08907 Barcelona, Spain; amunozf@bellvitgehospital.cat; 2 Department of Thoracic Surgery and Lung Transplantation, University Hospital Reina Sofía, 14004 Córdoba, Spain; pmoreno39@gmail.com (P.M.);; 3 Department of Pulmonology and Lung Transplantation, University Hospital Reina Sofía, 14004 Córdoba, Spain; 4 Department of Pathology and Experimental Therapeutics, University of Barcelona, 08036 Barcelona, Spain

**Keywords:** lung transplantation, airway complications, bronchial anastomosis, risk factors, outcomes

## Abstract

(1) Objective: To determine whether recent advances in lung transplantation (LT) have reduced the incidence and changed the risk factors for airway complications (AC). (2) Methods: Retrospective analysis of patients receiving a lung transplant between January 2007 and January 2019. An AC was defined as a bronchoscopic abnormality in the airway, either requiring or not requiring an endoscopic or surgical intervention. Both univariable and multivariable analyses were performed to identify risk factors for AC. (3) Results: 285 lung transplants (170 single and 115 bilateral lung transplants) were analysed, comprising 400 anastomoses at risk. A total of 50 anastomoses resulted in AC (12%). There were 14 anastomotic and 11 non-anastomotic stenoses, 4 dehiscences, and 3 malacias. Independent predictors for AC were: gender male (OR: 4.18; *p* = 0.002), cardiac comorbidities (OR: 2.74; *p* = 0.009), prolonged postoperative mechanical ventilation (OR: 2.5; *p* = 0.02), P_a_O_2_/F_i_O_2_ < 300 mmHg at 24 h post-LT (OR: 2.48; *p* = 0.01), graft infection (OR: 2.16; *p* = 0.05), and post-LT isolation of *Aspergillus* spp. (OR: 2.63; *p* = 0.03). (4) Conclusions: In spite of advances in lung transplantation practice, the risk factors, incidence, and lethality of AC after LT remains unchanged. Graft dysfunction, an infected environment, and the need of prolonged mechanical ventilation remain an *Achilles* heel for AC.

## 1. Introduction

Lung transplantation (LT) is an effective treatment option for selected recipients with end-stage lung diseases, offering an opportunity to improve quality of life and prolong survival. Despite the progress in organ preservation, surgical technique, and medical management of the recipient, airway complications (AC) remain a common problem after LT, contributing significantly to postoperative morbidity and mortality, with prevalence rates ranging from 2% to 18% in the most recent series [1,2].

The recognition and management of AC vary based on the time from transplant, location of the lesion, and severity. Unfortunately, most of the literature has not followed standardized definitions when analysing AC, explaining the high variability of incidence rates [1,2,3]. For this reason, the International Society of Heart and Lung Transplantation (ISHLT) has recently published a classification system aimed at allowing comparisons among transplant centers [1].

In our early experience analysing 151 bronchial anastmoses performed up to the year 2001, we reported a 5.3% rate of bronchial complications. In this initial series, complicated patients required longer periods of postoperative mechanical ventilation [4]. In 2008, we reassessed the issue considering changes in the transplant technique and perioperative management, observing a bronchial complication rate of 9%, and identifying airway colonizations, bilateral lung transplants, and longer postoperative mechanical ventilation as major risk factors for post-transplant AC [5].

In the last decade, there have been significant changes in our lung transplant activity, such as the unrestricted use of extended donors and donors after circulatory death, the ageing of the transplant candidates, the liberalization of ECMO, and changes in immunosuppression and perioperative management. Considering these changes, we aimed at reassessing the incidence of AC at our institution in a contemporary series and identifying potential changes in risk factors, following the standardized ISHLT classification of AC [1].

## 2. Materials and Methods

### 2.1. Patients

We reviewed 360 consecutive lung transplants performed at the University Hospital Reina Sofía in Córdoba (Córdoba, Spain) from January 2007 to January 2019. After excluding patients who did not survive more than one week after LT (deaths not related to AC) and recipients with major missing data, 285 patients were considered for the analysis (study group).

Similarly to our previous analyses [4,5], the unit of study was each bronchial anastomosis. Out of the 285 patients, 170 received a single lung transplant (SLT) and 115 received a bilateral lung transplant (BLT), comprising a total of 400 bronchial anastomoses at risk assessed over a median follow-up of 50 months (1 to 60 months).

### 2.2. Lung Transplant Procedure and Perioperative Management

The organ retrieval followed the standard technique of cardiopulmonary extraction [6]. Lungs were flushed with Perfadex^®^ (formulated as low-potassium dextran + glucose solution) [XVivo Gothenburg, Sweden], both antegradely prior to pulmonary retrieval and retrogradely on the back table [7]. 

A standard surgical procedure was followed to implant the lung grafts. Bronchial anastomoses were performed using a running end-to-end non-telescoping technique with an absorbable 4-0 monofilament polydioxanone suture [Ethicon, Sommerville, NJ, USA] without a tissue wrap and as close to the secondary carina as possible [8]. A standard running techinque was performed to complete both the pulmonary artery and left atrium anastomoses. After completion of the procedure, a bronchoscopy was performed to assess the airway and to aspirate secretions.

Postoperatively, we used a conventional maintenance immunosuppression that includes a calcineurin inhibitor, either tacrolimus [Prograf^®^; Fujisawa, Killorglin Co., Kerry, Ireland] or cyclosporine [Sandimmun^®^; Novartis, Basle, Switzerland]; an antiproliferative agent, either mycophenolate [Cellcept^®^; Roche Lab. Inc., Nutley, NJ, USA] or azathioprine [Imurel^®^; Medeva Pharma, Madrid, Spain]; and corticosteroids [Dezacor^®^; Hoechst Marion Roussel, Barcelona, Spain]. 

The antimicrobial strategy was based on antibiotic sensitivities from preoperative sputum cultures of the recipient and from the donor bronchoaspirate. Viral and fungal prophylaxis followed standardized protocols [9]. Those cases with bronchial fungal colonization, and those with AC treated bronchoscopically, received systemic voriconazole for 2 weeks and aerosolized amphotericin B for 3 months. In addition, oral nistatin was administered to cystic fibrosis recipients.

Induction therapy was used in some patients with an interleukin-2–receptor anti- body (IL-2) [Basiliximab^®^, Novartis, Basle, Switzerland] to minimize the onset of acute graft rejection episodes and to reduce the needs of maintenance immunosuppression. 

A scheduled bronchoscopic assessment of the airway was performed immediately after transplantation, before weaning and before hospital discharge. Additional bronchoscopies were performed whenever a clinical suspicion of infection or rejection arose.

Chronic lung allograft dysfunction (CLAD) was diagnosed either by transbronchial lung biopsy or on the basis of a decline in the forced expiratory volume in 1 s (FEV_1_), not related to other causes. Acute rejection episodes were identified either by transbronchial lung biopsy or by clinical criteria and were treated with methylprednisolone (10 mg/kg/day) for 3 consecutive days.

### 2.3. Endoscopic Management

The endoscopic treatment of AC included: dilation with rigid bronchoscopy, balloon dilation under flexible bronchoscopy, placement of Dumon silicone stents [Bryan, Woburn, MA, USA] or self-expandable metal stents [Ultraflex^®^, Boston Scientific, Galway, Ireland], and Nd-YAG laser resection for debridement of granulation tissue, as reported in our previous series [4,5].

### 2.4. Definitions

For the purposes of the present analysis, we followed the *ISHLT Consensus Statement on adult and pediatric airway complications after lung transplantation* [1], categorizing AC into anastomotic stenosis, non-anastomotic stenosis, malacia, and dehiscence. Importantly, as proposed by ISHLT classification, we have differentiated two types of bronchial stenosis: anastomotic stenosis (located at the bronchial anastomosis or within 2 cm) and non-anastomotic stenosis (affecting the airways distal to the anastomosis, most commonly at the bronchus intermedius). Airway malacia was defined as a 50% reduction in luminal calibre with expiration.

### 2.5. Data Collection, Statistical Analysis

All data were collected retrospectively. To identify predictors of AC, general demographic data, surgical, and other pre- and post-operative variables were recorded and possible associations between AC and potentially predictive clinical features were analysed by both univariable and multivariable tests. We conducted a multivariable logistic regression analysis to determine the odds ratios (OR) and the 95% confidence intervals (95% CI) for potential predictors of AC. Variables were selected when either a *p* value < 0.05 on univariable comparisons was observed or on the basis of their clinical relevance. Differences were considered significant for *p* values less than 0.05. The statistical analysis was performed using SPSS [IBM Corp. Released 2013. IBM SPSS Statistics for Windows, Version 22.0. Armonk, NY, USA: IBM Corp.].

## 3. Results

We reviewed 400 anastomoses at risk in 285 patients (170 SLT and 115 BLT). The mean age was 50 ± 9 years (18–67 years), corresponding to 216 (76%) males and 69 (24%) females. The preoperative diagnosis was emphysema (n = 134; 47%), interstitial lung disease (ILD) (n = 76; 27%), cystic fibrosis (CF) (n = 54; 19%), bronchiectasis (n = 6; 2%), and other indication in 15 (3%) patients.

### 3.1. Bronchial Complications

A total of 50 anastomoses presented AC (12.5%). Among them, 32 (8%) required either endoscopic or surgical intervention (Table 1); 14 (3.5%) anastomotic stenoses and 11 (2.7%) non-anastomotic stenoses required invasive management. Treatments included a combination of balloon dilation and ablative therapies (laser therapy, electrocautery, argon plasma coagulation, and cryotherapy) with or without application of mitomycin C. 

Asymptomatic malacia was present in 4 patients (1%), and it was managed conservatively, with non-invasive positive pressure ventilation. In 3 patients (0.7%) whose conservative treatment failed, an airway silicon stent was placed with excellent outcomes.

We have observed 6 anastomotic dehiscences (1.5%): 2 of them were treated conservatively without need of any intervention. The remaining 4 anastomoses—with more extensive necrosis—needed an invasive treatment: 3 of them were managed with a covered self-expanding metallic stent implanted with rigid bronchoscopy, and the remaining anastomotic dehiscence was managed with early surgical re-anastomosis with successful results. Unfortunately, 2 patients died secondary to their AC (0.5% of patients) (Table 1).

### 3.2. Univariable Analysis of Risk Factors

Recipient-related variables such as age, diagnosis, preoperative steroid use, or preoperative pulmonary hypertension have not been shown to be risk factors for AC. Only preoperative cardiac comorbidities (OR: 2.25; 95% CI: 1.15–4.37; *p* = 0.01) and gender male (OR: 2.3; 95% CI: 1.05–5.09; *p* = 0.03) were associated with the development of AC. Donor-related variables such as suboptimal donors have not been shown to be risk factors for AC (Table 2).

We have not identified intraoperative risk factors for AC by univariable analysis. On the contrary, several postoperative factors have been associated with AC: prolonged postoperative mechanical ventilation > 72 h (OR: 2.79; 95% CI: 1.48–5.20; *p* = 0.001), P_a_O_2_/F_i_O_2_ < 300 mmHg at 24 h post-lung transplantation (OR: 2.71; 95% CI: 1.44–5.09; *p* = 0.001), bronchial infection (OR: 3.25; 95% CI: 1.7–6.1; *p* < 0.001), and post-transplant isolation of *Aspergillus* spp. (OR: 2.16; 95% CI: 1.03–4.56; *p* = 0.03) (Table 2).

### 3.3. Multivariable Analysis of Risk Factors 

In the multivariable logistic regression model, we have included a selection of variables with statistical significance in the univariable analysis (*p* < 0.05) and some variables that we considered of clinical relevance. Only gender male (OR: 4.18; 95% CI: 1.68–10.4; *p* = 0.002), cardiac comorbidities (OR: 2.74; 95% CI: 1.28–5.84; *p* = 0.009), postoperative mechanical ventilation longer than 72 h (OR: 2.5; 95% CI: 1.15–5.3; *p* = 0.02), P_a_O_2_/F_i_O_2_ < 300 mmHg within the first 72 h after transplantation (OR: 2.48; 95% CI: 1.19–5.19; *p* = 0.01), bronchial infection (OR: 2.16; 95% CI: 1.0–4.7; *p* = 0.05), and post-transplant isolation of *Aspergillus* spp. (OR: 2.63; 95% CI: 1.10–6.29; *p* = 0.03) remained independent predictors for the development of AC (Table 3).

## 4. Discussion

Although in the early years of lung transplantation AC were a frequent cause of morbidity and mortality [8], the improvements in surgical and medical therapy have led to a significant decrease in these complications. Unfortunately, in the last two decades, the incidence of AC continues to be around 2% to 18% in large series [1,2,10]. Our experience confirms the stabilization of this incidence around 12% of all bronchial anastomoses, without significant changes in clinical risk factors. Bronchial ischemia due to factors such as primary graft dysfunction requiring long mechanical ventilation and an infected environment continue to be the major causes of bronchial ischemia-related AC.

The high variability in reported rates of AC is attributed to the different criteria to define AC. Recently, a group from Vienna reported an AC rate as low as 1.56% of 2941 anastomoses [11]. However, they only considered “relevant” AC requiring intervention. In our series, we have included all cases of bronchoscopic changes in the airway either requiring intervention or not. Moreover, this is the reason why our AC rate has risen to 12%. For this reason, the International Society for Heart and Lung Transplantation (ISHLT) developed a new classification to standardize the endoscopic findings based on the type of complication, its location, and its extent throughout the airway [1]. In the present series, we have followed the ISHLT classification to overcome the biases related to weak definitions of AC. 

Airway complications are usually related to donor airway ischemia and, frequently, one complication may lead to another with time. In the early phase, some degree of mucosal necrosis is observed. This may progress to involve the full thickness of the bronchial wall and produce an anastomotic dehiscence. Later on, these complications lead to fibrotic changes with bronchial stenosis, granulation tissue formation, and malacia [12].

Although some donor-related factors were reported to be associated with AC in the past [3], more recently, it has been demonstrated that extended donors with low PaO_2_/FiO_2_ and ventilated for a long time, did not negatively impact bronchial healing [13]. Similarly, we did not observe any specific donor factor to be involved in an impaired bronchial healing. This evidence has allowed us to broaden our lung donor pool, including those aged up to 70 years, with PaO_2_/FiO_2_ between 250 and 300 mmHg, or with a longer intubation time.

Deficient donor lung preservation impairs bronchial healing by decreasing the retrograde bronchial perfusion. The use of low-potassium dextran preservation solutions has allowed up to 12 h of lung preservation time [14]. In addition, we reported the benefits of a double anterograde and retrograde perfusion of the preservation solution to improve the post-transplant lung function [7].

The use of preoperative steroids is no longer a major concern regarding the airway healing. Furthermore, it has been reported that steroids are associated with less granulation tissue formation and longer survival [15]. As in our previous experience [4,5], in the present series, low doses of preoperative steroids were not related to AC and were proven to be useful in the prevention of acute rejection and potential amelioration of reperfusion injury.

The side and type of lung transplant may also have an influence on AC development. It has been demonstrated that right-sided bronchial anastomoses are associated with a higher risk of AC. This observation may be explained by the anatomical perfusion differences between both main bronchi. In the present series, we did not observe these differences. Of note that in our previous analysis, BLT were related to AC. In a series of 214 lung transplants, we observed that from 27 patients with AC, 23 were BLT, with a 7.4-fold risk for the development of bronchial complications than SLT [5]. In the present series, analysing 400 anastomoses, there were no differences regarding SLT vs. BLT. It is possible that fewer cases of cystic fibrosis patients transplanted in the last decade had an influence on these differences.

The anastomotic technique has evolved over time. Although a telescoped bronchial anastomosis was initially proposed as the best surgical technique in the early years [4], at present, the evidence favours the use of an end-to-end bronchial anastomosis with a single running suture, providing excellent results with a very low complication rate [11]. In the present series, all patients underwent either a double-running or a single-running suture with excellent results. In both cases, we performed an end-to-end technique with an absorbable 4/0 monofilament without tissue wrap, and the anastomosis was performed as close to the secondary carina as possible. We have not observed differences between both techniques, so we use both indistinctly with good results. Additionally, we have not identified differences in the rate of AC when recipient-donor bronchial size mismatch is evident. In such cases, we try to overcome this discrepancy by averaging and re-shaping both recipient and donor stumps.

Similarly to our previous experience [5], when postoperative variables were analysed, we observed that longer postoperative mechanical ventilation time, PaO_2_/FiO_2_ less than 300 mmHg during the first 72 h, severe postoperative graft infection, and post-transplant bronchial isolation of *Aspergillus* spp. were found to be associated with the presence of AC. These factors may be interrelated and seem to be consistent with the pathological processes known to be involved in the development of AC where the compromised blood flow is the suspected final common pathway [2,9,12].

Prolonged post-transplant mechanical ventilation carries positive pressure airflow in the airway, which might disrupt airway mucosal and anastomotic suture-line healing, contributing to the pathophysiology of AC. In our initial experience, recipients requiring long postoperative mechanical ventilation developed AC with a 3.5-fold risk higher than those being weaned from the ventilator early [5]. This association persisted in the present series. Factors making a long ventilator dependence necessary are most likely involved in the onset of AC, rather than the mechanical ventilation itself; these factors include PGD, infections, or hemodynamic instability, among others.

It has been demonstrated that bronchial infections impair anastomotic healing after transplantation. We observed more than two-fold higher risk of AC in those patients developing bronchial infections. Fungal infections, especially *Aspergillus*, are frequently present in ischemic anastomoses with a reported incidence around 24% [16], causing diffuse tracheobronchitis or infection at the anastomotic site. Similarly, in the present series, we demonstrated an association between airway colonization with *Aspergillus* spp. and the presence of AC.

Primary graft dysfunction has also been related to bronchial ischemia. These patients with diffuse alveolar damage and increased vascular permeability resulted in interstitial oedema and a reduction in pulmonary blood flow [17]. Although we were not able to demonstrate an association between AC and patients developing PGD, when analysing recipient PaO_2_/FiO_2_ within 72 h post-transplant, those patients below 300 mmHg were at 2.4 higher risk of developing AC.

In the present series, recipient male gender was an independent risk factor for AC. Previous studies [10] have observed the length of the recipient being an independent risk factor for AC. Additionally, right-sided bronchial anastomoses (having a wider diameter than left-sided ones) have been reported to be associated with AC. Whether there are gender-related anatomic changes in the airway that make males more prone to AC is the subject of further investigation.

Bronchial dehiscence is a major complication associated with high mortality rates [1,2,10,12]. It is the consequence of the natural progression of a mucosal necrosis within the first 1–4 weeks after the lung transplant. The reported incidence ranges between 1 and 10%. Mild partial dehiscences are best managed conservatively. A step forward is the insertion of a stent during 6–8 weeks after the anastomotic healing has been completed. If self-expanding metallic stents are used to manage the dehiscence, it is frequently the development of subsequent granulation tissue that may require further debridement [18,19].

Complete dehiscences are fatal complications associated with high mortality rates related to infectious complications and ventilatory problems. Usually, these patients present lung collapse despite an adequate drainage of the pleural space and invasive mechanical ventilation. Open surgical repair, flap bronchoplasty, or transplantectomy are therapeutic options with poor outcomes [20]. 

Among six dehiscences in the present series, two patients died as a consequence of complete dehiscence secondary to bronchial infection that progressed to sepsis and multiorganic failure. The remaining cases were treated successfully with conservative measures, stent implantation, or re-anastomosis.

Bronchial stenosis is the most frequent AC with an estimated incidence ranging from 5% to 30% [1,2,10,12]. It is the natural consequence of a dehiscence or infection appearing within 2 to 9 months after the lung transplant. Repeated balloon bronchial dilatations are the first therapeutic option; however, these are generally insufficient to resolve moderate to severe stenoses. In such cases, combined procedures including debridement of necrotic tissue and electrocautery resection with or without local injection of steroids and mitomycin-C are required [21]. Covered or uncovered self-expanding metallic stents are the best option to maintain the patency of the airway with immediate relief of dyspnea. When endoscopic procedures fail, especially for non-anastomotic stenoses, surgical options include bronchial anastomosis reconstruction, sleeve resections, or retransplantation [22]. In our experience, these procedures are extremely unusual, but the possibility of a surgical resection for those patients with distal AC complicating the lung parenchyma is an option to be considered [5].

Malacic airways are frequent in lung recipients and in those stenotic airways treated with stents. In the present series, only three patients required the insertion of a self-expandable metal stent with satisfactory results.

The present study had several limitations. First, the study was retrospective, and therefore, only historical comparisons were conducted. Second, patients were subject to some surgical variability over time. Third, methods of lung preservation, immunosuppression, and postoperative management have evolved throughout the study period. Fourth, the assessment of risk factors in the clinical setting is challenging and demonstrating whether a single factor is a cause or a consequence of another is difficult. Finally, although we followed the ISHLT classification of airway complications, the severity of AC was based on the subjectivity of the bronchoscopist.

## 5. Conclusions

To summarize, the incidence of airway complications after lung transplantation has remained unchanged in the last decade. Recipients with post-transplant graft dysfunction and infections needing prolonged mechanical ventilation are the main factors leading to graft ischemia and, ultimately, an airway complication. The majority of cases are best managed with endoscopic procedures, but, on ocassion, a surgical revision may be necessary to resolve these complications.

## Figures and Tables

**Table 1:** Incidence and general management of airway complications (AC) after lung transplantation in the present series.

Total AC n = 50 (12.5%)	Intervention n = 32 (8%)	No Intervention n = 18 (4.5%)	Deaths n = 2 (0.5%)
Anastomotic stenosis	14 (3.5%)	6 (1.5%)	0
Non-anastomotic stenosis	11 (2.7%)	5 (1.2%)	0
Malacia	3 (0.7%)	4 (1%)	0
Dehiscence	4 (1%)	2 (0.5%)	2 (0.5%)

**Table 2:** Variables associated with airway complications (AC) by univariable analysis (anastomosis-based analysis).

	OR (95% CI)	*p*
*Recipient-related independent variables*		
Sex _male_	2.30 (1.05–5.09)	0.03
Age _> 55 years_	1.15 (0.64–2.09)	0.63
Cardiac comorbidities _YES_	2.25 (1.15–4.37)	0.01
Previous pulmonary hypertension _YES_	1.39 (0.66–2.92)	0.38
Perioperative steroid use _YES_ Disease	0.62 (0.20–1.32)	0.21
● Emphysema	0.96 (0.53–1.75)	0.90
● Cystic fibrosis	0.86 (0.43–1.72)	0.67
● Pulmonary fibrosis	0.79 (0.34–1.85)	0.60
● Bronchiectasis	0.62 (0.08–4.93)	0.54
● Other	1.96 (0.88–4.36)	0.09
*Donor-related independent variables*		
Suboptimal donors	1.64 (0.85–2.25)	0.13
*Intraoperative variables*		
Type of Transplantation _BLT_	0.79 (0.44–1.44)	0.45
Anastomotic side _right_	1.23 (0.66–2.28)	0.51
Incision _clamshell_	1.09 (0.58–2.04)	0.78
CEC/ECMO _YES_	1.82 (0.91–3.65)	0.08
D/R bronchial size mismatch _YES_	0.94 (0.47–1.88)	0.87
Suture _conventional_	3.04 (0.71–12.99)	0.11
*Postoperative variables*		
Induction therapy _No induction_	1.05 (0.54–2.03)	0.88
P_a_O_2_/F_i_O_2_ < 300 _YES_	2.71 (1.44–5.09)	0.001
Primary Graft Dysfunction _YES_	1.71 (0.91–3.19)	0.09
Acute graft rejection _YES_	1.08 (0.52–2.28)	0.83
Mechanical ventilation > 72 h _YES_	2.79 (1.48–5.20)	0.001
Bronchial Infection _YES_	3.25 (1.70–6.10)	<0.001
Bleeding _YES_	1.84 (0.71–4.74)	0.20
CLAD _YES_	0.69 (0.28–1.70)	0.41
*Microbiome isolations after lung transplantation*		
*Candida* spp.	0.67 (0.23–1.90)	0.45
*Aspergillus* spp.	2.16 (1.03–4.56)	0.03
*Burkhodelia* spp.	1.01 (0.22–4.59)	0.99
*A. Xyloides*	0.32 (0.40–2.40)	0.24
*Escherichia* spp.	1.15 (0.25–5.32)	1.15
*Enterobacter* spp.	3.50 (0.63–19.90)	0.12
*Haemophilus* spp.	1.12 (0.37–3.36)	0.84
*Klebsiella* spp.	2.85 (0.54–15.10)	0.20
*Tuberculosis* spp.	1.59 (0.81–3.12)	0.17
*Pseudomona* spp.	1.60 (0.89–2.94)	0.11
*Staphilococus* spp.	1.07 (0.55–2.09)	0.82
*Serratia* spp.	0.99 (0.20–3.45)	0.98
*Streptomona* spp.	0.90 (0.20–4.15)	0.92

BLT: Bilateral lung transplantation CEC: Conventional extracorporeal circulation; CLAD: Chronic lung allograft dysfunction; D/R: Donor/Recipient.

**Table 3:** Independent risk factors for airway complications (AC) after lung transplantation by multivariable logistic regression analysis.

Variables	OR	95% CI	*p* Value
Gender _male_	4.18	1.68–10.40	0.002
Cardiac comorbidities	2.74	1.28–5.84	0.009
Mechanical Ventilation > 72 h postop.	2.50	1.15–5.30	0.02
P_a_O_2_/F_i_O_2_ < 300 at 72 h postop.	2.48	1.19–5.19	0.015
Primary Graft Dysfunction	0.88	0.38–2.05	0.77
Acute cellular rejection	0.84	0.35–1.96	0.68
Bronchial Infection	2.16	1.0–4.70	0.05
Bleeding	2.23	0.74–6.65	0.15
*Aspergillus* spp. bronchial isolation	2.63	1.10–6.29	0.03

## Data Availability

Due to patients’ privacy and ethical restrictions, an anonymized dataset can be shared on reasonable request to the corresponding author.
